# Application of Neurally Adjusted Ventilatory Assist in Premature Neonates Less Than 1,500 Grams With Established or Evolving Bronchopulmonary Dysplasia

**DOI:** 10.3389/fped.2020.00110

**Published:** 2020-03-24

**Authors:** Xiao Rong, Feng Liang, Yuan-Jing Li, Hong Liang, Xiao-Peng Zhao, Hong-Mei Zou, Wei-Neng Lu, Hui Shi, Jing-Hua Zhang, Rui-Lian Guan, Yi Sun, Huayan Zhang

**Affiliations:** ^1^Division of Neonatology, Guangzhou Women and Children's Medical Center Affiliated With Jinan University, Guanghzou, China; ^2^Division of Neonatology, Guangzhou Women and Children's Medical Center, Guanghzou, China; ^3^Children's Hospital of Philadelphia and University of Pennsylvania Perelman School of Medicine, Philadelphia, PA, United States

**Keywords:** premature infants, bronchopulmonary dysplasia, BPD, neurally adjusted ventilatory assist ventilation, NAVA, very low birth weight, VLBW, mechanical ventilation

## Abstract

**Background:** Very low birth weight premature (VLBW) infants with bronchopulmonary dysplasia (BPD) often need prolonged respiratory support, which is associated with worse outcomes. The application of neurally adjusted ventilatory assist ventilation (NAVA) in infants with BPD has rarely been reported. This study investigated whether NAVA is safe and can reduce the duration respiratory support in VLBW premature infants with established or evolving BPD.

**Methods:** This retrospective matched-cohort study included patients admitted to our NICU between April 2017 to April 2019 who were born at <32 weeks' gestation with birthweight of <1,500 g. The study groups (NAVA group) were infants who received NAVA ventilation as a sequel mode of ventilation after at least 2 weeks of traditional respiratory support after birth. The control group were preterm infants who required traditional respiratory support beyond first 2 weeks of life and were closely matched to the NAVA patients by gestational age and birthweight. The primary outcome was to compare the total duration of respiratory support between the NAVA group and the control group. The secondary outcomes were comparisons of duration of invasive and non-invasive support, oxygen therapy, length of stay, severity of BPD, weight gain and sedation need between the groups.

**Results:** There were no significant differences between NAVA group and control group in the primary and most of the secondary outcomes (all *P* > 0.05). However, NAVA was well tolerated and there was a decrease in the need of sedation (*p* = 0.012) after switching to NAVA.

**Conclusion:** NAVA, when used as a sequel mode of ventilation, in premature neonates <1,500 g with evolving or established BPD showed a similar effect compared to conventional ventilation in respiratory outcomes. NAVA can be safely used in this patient population and potentially can decrease the need of sedation.

## Introduction

Bronchopulmonary dysplasia (BPD), a chronic neonatal lung disease, is among the most common and severe sequelae of preterm birth. Despite improvements in neonatal care over the past 30 years, BPD rates have not declined. Stoll et al. reported an overall incidence of BPD in infants born 22–28 weeks' gestation age (GA) and 401–1,500 g birthweight increased from 36% in 1993–1997 to 45% in 2008–2012 in the United States (1). In a recently published study, Lui et al. also reported increased incidence of BPD in very low birthweight infants (VLBW born at <1,500 g) and <32 weeks' GA over time in most of the 11 high-income countries participating in the International Network for Evaluating Outcomes (iNeo) of neonates. The overall rate of BPD in the iNeo network increased from 23.3% in 2007–2011 to 27.5% in 2012-2015 (2). BPD predisposes survivors to adverse neurodevelopment and cardiorespiratory health and is associated with substantial resource utilization and cost (3, 4). Unfortunately, there are few evidence-based therapies to prevent and treat the disease (1, 5, 6). Infants with BPD often require prolonged respiratory support, and some need extended duration of intubated mechanical ventilation. Current data suggest that both prolonged mechanical ventilation and respiratory support of any type is associated with poor outcomes in extremely low birthweight infants (7, 8). Therefore, decreasing the total duration of respiratory support, especially invasive mechanical ventilation is important in improving the outcomes of preterm infants.

Neurally adjusted ventilatory assist ventilation (NAVA) is a new form of ventilation developed in the recent years. In this form of ventilation, ventilatory support is initiated when an electrical signal from the diaphragm muscle is detected by a probe placed in the distal esophagus. The level of inspiratory pressure provided is synchronized and in proportion to the electrical activity of the diaphragm (EAdi) (9, 10). Compared to traditional ventilation with pressure or flow triggering mechanisms, this mode of ventilation may provide better synchronized breath and more precise amount of support that fits the patients' needs without sedation. NAVA has been considered to be one of the gentlest ventilation modes available and by improving patient-ventilator synchrony, it may potentially reduce ventilator induced lung injury, and decrease sedation use in preterm infants (11–13). However, data on the safety and efficacy of NAVA ventilation in the VLBW infants, especially in infants with evolving or established BPD are limited. In this study, we aimed to test the hypothesis that NAVA ventilation reduced the duration of respiratory support in VLBW infants with evolving or established BPD and examine the safety of NAVA use in this population.

## Materials and Methods

This retrospective matched-cohort study was performed in the Neonatal Intensive Care Unit (NICU) of Guangzhou Women and Children's Medical Center, China between April 2017 to April 2019. The study cohort included preterm infants born at <32 weeks' gestational age (GA) and birthweight < 1,500 g with evolving or established BPD, who were switched to NAVA ventilation from invasive or non-invasive ventilation beyond 2 weeks of life. A comparison cohort was selected by 1 to 1 matching to the NAVA patient using the following matching criteria: (1) preterm infants born at <32 weeks' GA and birthweight < 1,500 g who were admitted to our NICU during the study period; (2) matching to a patient in the NAVA group first by similar GA (within 10 days), and then by similar birthweight (within 250 g); (3) required respiratory support of CPAP or higher (BiPAP, NIPPV, or intubated mechanical ventilation) for more than 2 weeks after birth and remained on traditional respiratory support modes during the hospital stay. Infants who had congenital anomalies and who were transferred from another hospital to undergo surgery were excluded.

NAVA or NIV-NAVA was provided by the SERVO-n (Maquet Critical Care AB, Solna, Sweden) ventilator system with the NAVA option. When switching from conventional mechanical ventilation to NAVA, the NAVA level was changed to match the peak inspiratory pressures delivered during the previous conventional ventilation. During the NAVA or NIV-NAVA support, the NAVA level was adjusted, based on EAdi (EAdi peak between 5 and 15 μV), transcutaneous carbon dioxide (tcPCO_2_) monitoring and blood gas analyses. PEEP was initially set at the same level as the previous ventilator PEEP and then adjusted based on chest X-ray findings to avoid hyperinflation. Settings similar to previous ventilator settings were used as the backup settings, with the “apnea time” set at 2–4 s initially and modified based on the severity of apnea. Back up ventilation will start when the ventilator could not detect the EAdi for more than the “apnea time” (set by the caregiver). When the ventilator detected a sufficient EAdi signal, the NAVA ventilation would resume. Weaning from NAVA support was done by decreasing the NAVA level and prolonging the apnea time in a step-wise manner as tolerated without clinical deterioration. When the NAVA level was <1, and the apnea time was more than 6 s, we would extubate and change to NIV-NAVA, nasal continuous positive airway pressure (nCPAP) or biphasic positive airway pressure (BiPAP) or wean to oxygen, according to the decision of the physician on duty. When changing from invasive NAVA mode to NIV-NAVA mode, the NAVA level would be increased to 1.5–2.0 and adjusted based on the EAdi (EAdi peak between 5 and 15 μV), tcPCO2 monitoring and/or blood gas analyses, with the same PEEP level, the “apnea time” would be decrease to 4 s, and FiO_2_ adjusted by the SPO_2_ target. To wean from NIV-NAVA, we would again decrease the NAVA level and prolong the apnea time in a step-wise manner as tolerated. When the NAVA level was <0.5, and the apnea time was more than 10 s, we would change to nCPAP or BiPAP or wean to oxygen, according to the decision of the physician on duty.

The primary outcome was to compare the total duration of respiratory support between the NAVA group and the control group. Secondary outcomes examined include severity of BPD, duration of invasive and non-invasive ventilation, duration of total oxygen, rate of home oxygen therapy (HOT), length of hospital stay, weight gain, medications use for the treatment of BPD, duration of sedation requirements, as well as major complication during the NICU stay. Major complications assessed included the incidences of necrotizing enterocolitis (NEC), late onset sepsis (LOS), intraventricular hemorrhage (IVH)/periventricular leukomalacia (PVL), retinopathy of prematurity (ROP), and patent ductus arteriosus (PDA).

BPD and severity of BPD were defined based on the NICHD consensus definition: infants are diagnosed to have BPD if they received an accumulative oxygen therapy of at least 28 days after birth; and at 36 weeks' postmenstrual age (PMA), to have mild BPD if breathing room air, moderate BPD if on <30% oxygen, and severe BPD if on at least 30% oxygen or on positive pressure support (12). Respiratory severity scores (RSS) were calculated using mean airway pressure (MAP) x inspired oxygen concentration (FiO_2_). RSS has been shown to reflect the severity of respiratory illness and correlated well with an oxygenation marker, the oxygen index (OI) in newborn infants (13). RSS was calculated on admission and again on the day NAVA support was started for the infants in the NAVA group and their matched comparison patient. For example, if an infant in the NAVA group was changed to NAVA on day of life 29, RSS was calculated for that infant based on the MAP and FiO_2_ requirement in the morning that day before changing to NAVA. The RSS for his/her matching comparison patient was calculated based on the level of support on day of life 29.

“Sedation use day” in this study was defined as the total number of days during which a sedative or analgesic medication (i.e., midazolam, fentanyl, or morphine) was used. The sedation/analgesia policy in our NICU has been evolving over time. Before October 2018, Midazolam was the first-choice sedative when the preterm infants showed frequent desaturation and retractions due to agitation with supplemental oxygen needs over 40%. Fentanyl drip would be added if the infants still shows signs of agitation with midazolam up to 0.2 mg/kg.h. After October 2018, morphine has become the first-line chronic analgesia/sedation choice for infants on mechanical ventilation in our unit. Midazolam would be added when the infant was considered needing more sedation on morphine dose of 0.06 mg/kg.h. The doses of these medication would be gradually weaned when the infant was clinically improving and weaning on the ventilator support. Fentanyl is routinely administered before invasive procedures, such as thoracentesis, paracentesis, and chest tube placement.

The use of common medications frequently used in infants with evolving or established BPD were recorded. These medications included corticosteroid, diuretics and bronchodilators. In addition, the total accumulative dose of dexamethasone per kilogram body weight was also recorded. Indications for the use of indications were at the discretion of the treating physician and may include steroid use to facilitate extubation or weaning on ventilator support, diuretic use to decrease pulmonary edema and bronchodilator use for clinical wheezing.

In addition to comparing the NAVA patients with the matched comparison group. Patients in the NAVA group also served as self-controls. The weight gain velocity and the days on sedation/analgesia medication before and after starting on NAVA support were compared in the NAVA group. All data were abstracted from patients' medical records and the Institutional Review Board of Guangzhou Women and Children's Medical Center approved this study.

### Statistical Analysis

Demographic data were summarized with standard descriptive statistics. For continuous measurement data, normal distribution variables were analyzed by *t*-test, and non-normal distribution variables were analyzed by Wilcolxon rank-sum test. Categorical variables between groups were analyzed using chi-square test. All statistical analyses were performed using SPSS Statistics version 22.0 (SPSS Inc., Chicago, IL, United States). In addition, for all statistical analyses executed, we considered a two-tailed *p*-value of <0.05 to be statistically significant.

## Results

During the two-year period, 15 preterm infants who were born at <32 weeks' GA and <1,500 g received NAVA ventilation. 15 infants who received traditional modes of respiratory support during the same period were selected by matching 1:1 to the NAVA patients according to the matching criteria. Table 1 presents the basic characteristics of the NAVA group and the matched comparison group. There was no significant difference between the two groups.

**Table 1:** Basic characteristics of the study groups.

	**Groups**		***p***
	**NAVA** ***n* = 15**	**Control** ***n* = 15**	
Basic demographics			
Gestational age, week ± SD^*^	28.1 ± 1.4	28.0 ± 1.7	0.881
Birth weight g ± SD^*^	965.33 ± 217.48	998.67 ± 185.62	0.655
Gender, Male/Female	11/4	10/5	0.690
Cesarean section, *n* (%)	11 (73.3)	11 (73.3)	1.000
Antenatal steroids, *n* (%)	9 (81.8)	7 (63.6)	0.464
Apgar at 1 min, median (Q1, Q3) Apgar at 5 min, median (Q1, Q3)	8 (4,9) 8 (7,9)	8 (6,8) 8 (8,9)	0.680 0.949
Severity of lung disease			
RSS on admission^*^	3.59 ± 1.37	3.63 ± 1.41	0.942
RSS when changed to NAVA mode^*^	2.52 ± 0.80	2.02 ± 1.93	0.369
Severity of BPD			
Moderate/severe	7/8	12/3	0.058

*RSS, respiratory severity score. Normal distribution variables were presented as mean ± SD, non-normal distribution variables were presented as median (quartile range). Normal distribution variables were analyzed by t-test, and non-normal distribution variables were analyzed by Wilcolxon rank-sum test*.

* *Analyzed by t-test, others analyzed by Wilcolxon rank-sum test*.

The patients in the NAVA group were transferred to NAVA support at a median time of 34 days (quartile range 29, 39 days) after birth. Out of the 15 patients, 3 were started on NAVA prior to 28 days of life (on day of life 17, 25, and 27) and therefore considered to have evolving BPD at the time. Indications for transferring to NAVA included prolonged need of invasive or non-invasive ventilation beyond first 2 weeks of life and projected to not able to wean from ventilation within a short period of time. There were 12 patients changed from synchronized intermittent mandatory ventilation (SIMV) to NAVA invasive ventilation. Among these 12 patients, 10 were successfully extubated to either NIV-NAVA or nasal continuous positive airway pressure (nCPAP) or bi-level positive airway pressure (BiPAP) support. Two patients had clinical deterioration and need to convert to high frequency oscillatory ventilation (HFOV) or back to SIMV. However, both were on NAVA for more than 72 h (3 days and 8 days). Two patients directly extubated from SIMV mode to NIV-NAVA mode, then weaned to nCPAP. One patient who was on non-invasive ventilation for more than 28 days was changed from BiPAP mode to NIV-NAVA, and then successfully weaned to low flow oxygen.

There were no significant differences between the NAVA group and the control group in the primary outcome of total duration of respiratory support and most of the secondary outcomes including duration of invasive ventilation, duration of non-invasive ventilation, duration of oxygen therapy, length of hospital stay, severity of BPD, weight gain, or total days on sedation medications (all *p* > 0.05, Table 2). All patients in this study had moderate to severe BPD. In the NAVA group, patients were transferred to NAVA support on median GA of 33 weeks (quartile range 32, 35). Twelve of the fifteen patients in this group can be diagnosed with BPD at the time of starting on NAVA. However, only one patient reached 36 weeks PMA and could be diagnosed with severe BPD at the time. Although there was a trend towards more severe BPD in the NAVA group, it did not reach statistical significance.

**Table 2:** Comparison of primary and secondary outcomes between the two groups.

	**Groups**		***p***
	**NAVA**	**Control**	
	***n* = 15**	***n* = 15**	
Total duration of respiratory support, days ± SD	60.4 ± 19.2	59.5 ± 26.3	0.867
Duration of invasive respiratory ventilation, days ± SD	35.7 ± 18.3	29.1 ± 23.2	0.40
Duration of non-invasive ventilation,days ± SD	24.7 ± 12.9	30.4 ± 15.0	0.277
Duration of oxygen therapy, days ± SD	76.1 ± 18.8	69.2 ± 22.1	0.362
Length of hospital stay,days ± SD	84.1 ± 21.2	83.9 ± 19.7	0.979
Weight gain, gram/d	19.9 ± 5.5	19.3 ± 5.4	0.744
Sedation use day, median (Q1, Q3)	17.0 (0.0, 39.0)	6.0 (3.0, 32.0)	0.683
	**NAVA group**		***p***
	**Before NAVA**	**After NAVA**	
Weight gain, gram/d	17.2 ± 6.1	20.4 ± 8.9	0.258
Sedation use day, median (Q1, Q3)	17.0 (0.0, 38.0)	0.0 (0.0,0.0)	0.012^*^

* *There were significant decrease in the need of sedation after changing to NAVA ventilation in the NAVA group*.

However, there were significant decrease in the need of sedation after changing to NAVA ventilation in the NAVA group (Table 2). Of the 15 patients in the NAVA, only the two patients who needed to convert back to SIMV or HFOV continued to required sedation. One patient on chronic morphine therapy before switching to NAVA weaned to a tapering dose of enteral morphine and the rest of 12 patients did not required any sedation after switching to NAVA.

Overall, NAVA ventilation was well tolerated without significant events and the rate of the common complications in the premature infants, including necrotizing enterocolitis, LOS, intraventricular hemorrhage/periventricular leukomalacia, ROP, and PDA were comparable between the two groups (all *P* > 0.05, Table 3). Medication therapy for BPD treatment and the rate of HOT were also similar between the two groups (Table 4).

**Table 3:** Comparison of complications of premature infants between the two groups.

**Complications**	**Groups**		***p***
	**NAVA** ***n* = 15 (%)**	**Control** ***n* = 15(%)**	
NEC	1 (6.7)	3 (20.0)	0.283
IVH/PVL	6 (40.0)	6 (40.0)	1.000
LOS	12 (80.0)	8 (53.3)	0.121
ROP	13 (86.7)	10 (66.7)	0.195
ROP required surgical treatment	1 (6.7)	4 (26.7)	0.142
PDA	9 (60.0)	9 (60.0)	1.000
PDA required medical treatment	7 (46.7)	7 (46.7)	1.000
PDA required ligation	2 (13.3)	4 (26.7)	0.361

*NEC, necrotizing enterocolitis; IVH, intraventricular hemorrhage; PVL, periventricular leukomalacia; LOS, late onset sepsis; BPD, bronchopulmonary dysplasia; ROP, retinopathy of prematurity; PDA, patent ductus arteriosus*.

**Table 4:** Comparison of medical treatments for BPD between the two groups.

	**Groups**		
	**NAVA** ***n* = 15**	**Control** ***n* = 15**	***p***
Medical therapies of BPD			
Corticosteroid (%)	5 (33.3%)	7 (46.7%)	0.456
Total amount of Dexamethasone(mg/kg)	1.47 ± 0.66	1.19 ± 0.40	0.381
Diuretics (%)	6 (40.0%)	8 (53.3%)	0.464
Bronchodilators (%)	6 (40.0%)	10 (66.7%)	0.143
HOT (%)	3 (20.0%)	4 (26.7%)	0.666

*Dexamethasone dose was the total accumulative dose of the medication. HOT, home oxygen therapy*.

## Discussion

This study examined the utility of NAVA ventilation in premature infants with evolving and established BPD. Infants were switched to NAVA ventilation after being on conventional mechanical ventilation or high-level non-invasive ventilation for extended period of time (median time to NAVA 34 days). Although the NAVA group and the control group in this study were similar in the basic demographics, there was a trend toward more severe BPD in the NAVA group (8/15 vs. 3/15), which did not reach statistical significance. This could suggest that NAVA was used in patients with more severe lung disease. Despite this, transition to NAVA ventilation was well tolerated in all patients with no complications.

There have been limited data available regarding the use of NAVA in the neonates. Most of these studies examined the very short-term effects of NAVA. Some reported improved patient-ventilator interaction, or decreased PIP within 24 h of NAVA use (10, 14). Oda et al. reported NAVA use in 14 extremely low birth weight infants. There was no difference in the incidence of BPD, HOT or the duration of intubation when the NAVA group was compared to a historical control of 21 ELBW patients before the implementation of NAVA. Although no difference was found in the total duration of sedation use, midazolam was discontinued in all patients after switching to NAVA (15). We found similar result in our study that there was no difference in the total duration of sedation use, but the NAVA group had significantly decreased sedation use after switching to NAVA.

Very few studies have reported NAVA use in infants with evolving or established BPD. In a crossover study, Shetty et al. enrolled 9 premature infants with evolving or established BPD and reported lower oxygen index, FiO_2_ requirement, PIP and MAP after switching to NAVA from assist control ventilation for 1 h (16). Jung et al. reported decreased RSS and ventilator variables within the first 24 h of switching from SIMV to NAVA (17). However, neither of these studies reported outcomes beyond 24 h of use of NAVA. Lee et al. examined the use of NAVA in 9 infants with severe BPD who were on chronic mechanical ventilation via tracheostomy and compared to 5 similar infants on pneumatically triggered ventilation. They found decreased cyanotic episodes, as well as reduced need for sedatives and dexamethasone (18). Our study focused on infants with evolving and established BPD and reported outcomes at NICU discharge as compared to GA and birthweight matched controls with BPD. We found no difference in the duration of respiratory support, HOT or length of stay. However, our study demonstrated that NAVA use was safe in this patient population and associated with decreased sedation needs after being on NAVA. This was probably because NAVA allowed for the patient to trigger the ventilator easier and faster than on the conventional ventilator triggering mechanism. The better patient-ventilator synchrony therefore making them more comfortable on the ventilator and less agitated. With the concerns that prolonged and high dose sedation may have negative effects on the long-term neurodevelopmental outcomes of very low birthweight preterm infants, decreased need for sedation on NAVA support may have longer-term benefits in this population.

There are several limitations of this study. First, with the retrospective nature of the study, there might be a variety of confounders that could influence the outcome of the study. The small sample size makes the statistical power low. The negative findings of study could therefore result from type II error due to the small sample size. Increasing the number of patients in the comparison group by matching two patients to one NAVA patient might be able to increase the statistical power. Unfortunately, we were unable to find enough patients who were on respiratory support of CPAP or higher for more than 2 weeks after birth that were also closely matched in both GA and BW to the NAVA patients, to enable this 2 to 1 matching. Second, it was very hard to find an optimal control group. We have tried to select patients that were as closely matched to the NAVA group as possible. Although the basic demographics and RSS were similar between the two groups, there was a non-statistically higher number of patients with severe BPD in the NAVA group, which could have been a result of selection bias. This could also contribute to the finding of no difference in the primary outcome of the study. Third, the criteria for the application of NAVA had not been established. Variations in the use of NAVA ventilation and medications could also affects the outcomes. Luckily, the staffing of medical team was stable and there was no significant variation in the medical management between different patients during the study period. Despite these limitations, our study provided more data for the utility of NAVA in patients with BPD.

## Conclusion

NAVA, when used as a sequel mode of ventilation, in premature neonates born at <1,500 g with established or evolving BPD showed a similar effect compared to conventional ventilation in respiratory outcomes. NAVA can be safely used in this patient population and potentially can decrease the need of sedation. Prospective studies with larger sample size are needed to delineate the effect of NAVA in patients with BPD.

## Data Availability Statement

The datasets generated for this study are available on request to the corresponding author.

## Ethics Statement

The studies involving human participants were reviewed and approved by Research ethics committee of Guangzhou Women and Children's Medical Center. Written informed consent from the participants' legal guardian/next of kin was not required to participate in this study in accordance with the national legislation and the institutional requirements.

## Author Contributions

XR made substantial contributions to the conception and design of the study, acquisition of data, and drafted the initial manuscript. FL participated in the design of the study, performed all the data analysis, and approved the draft of the manuscript. Y-JL, HL, X-PZ, H-MZ, W-NL, HS, J-HZ, R-LG, and YS participated in data acquisition and approved the draft of the manuscript. HZ developed the original study design and protocol, supervised the analyses, interpreted the results, and critically revised all drafts of the manuscript including the final manuscript.

### Conflict of Interest

The authors declare that the research was conducted in the absence of any commercial or financial relationships that could be construed as a potential conflict of interest.
